# Postnatal expression of the transcription factor Ebf2 in motivation, reward, and pain-related circuits of the mouse brain

**DOI:** 10.3389/fnana.2026.1780361

**Published:** 2026-03-04

**Authors:** Moisés Martínez-Estrada, M. Blanca D. Cepeda-Varela, A. Damaris Salinas-Villarreal, Mara C. Obregón-Fuentes, Danna A. Real-Marín, Melani A. Balderas-Díaz, Viviana Zomosa-Signoret, Jesús Santana-Solano, Moisés Santillán-Zerón, Román Vidaltamayo

**Affiliations:** 1 Centro de Investigación y de Estudios Avanzados del IPN, Unidad Monterrey, Apodaca, Mexico; 2 Facultad de Ciencias en Física y Matemáticas, Universidad Autónoma de Chiapas, Tuxtla Gutiérrez, Mexico; 3 Facultad de Medicina, Universidad de Monterrey, San Pedro Garza García, Mexico; 4 Escuela de Ciencias Aliadas de la Salud, Universidad de Monterrey, San Pedro Garza García, Mexico

**Keywords:** early B-cell factor, Ebf2 gene, motivation/reward system, pain-modulating systems, transcription factor Ebf2

## Abstract

**Introduction:**

Early B-cell factor 2 (Ebf2) is a transcription factor required for neuronal differentiation. However, its postnatal expression pattern and functional roles in the brain are not well characterized. This study examined the spatial distribution of Ebf2 in postnatal day 10 (P10) mouse brains and investigated its association with neural circuits mediating motivation, reward, and nociception.

**Materials and methods:**

Ebf2-TGFP transgenic mice, which express green fluorescent protein (GFP) as a reporter for Ebf2, were utilized. Immunofluorescence labeling and high-resolution microscopy were employed to visualize Ebf2 expression. Image data were analyzed using a deep learning–based segmentation pipeline for soma and axon identification. Three-dimensional reconstructions were registered to the Allen Brain Atlas. Quantitative comparisons between hemizygous and Ebf2-null mutant genotypes were conducted using linear mixed-effects models with Bonferroni and false discovery rate (FDR) corrections.

**Results:**

Ebf2 expression was prominent in the dorsal diencephalic conduction system, including the septum, habenula, and interpeduncular nucleus. Ebf2 expression can also be detected in the lateral hypothalamic area, zona incerta, ventral tegmental area, and parabrachial nucleus. Expression was also detected in nociceptive and sensory-motor regions such as the periaqueductal gray, anterior pretectal nucleus, principal sensory nucleus of the trigeminal nerve, and superior colliculus. Ebf2-null mutant mice showed a significant reduction in Ebf2-TGFP cells across most of these regions.

**Discussion:**

The results demonstrate that Ebf2 expression persists beyond embryonic development and is selectively enriched in neural circuits associated with motivation, reward processing, and nociceptive modulation. The marked reduction of Ebf2-TGFP expressing neurons in null mutants provides evidence for a postnatal requirement of Ebf2 in neuronal maintenance, rather than solely in early differentiation. Collectively, these findings broaden the functional scope of Ebf2 to include postnatal circuit stabilization and support its sustained regulatory role in brain systems that govern affective and pain-related behaviors.

## 1 Introduction

The family of early B cell factor (Ebf) genes encodes a homologous group of helix–loop–helix transcription factors highly conserved in evolution, consisting of four members in mammals (Wang et al., 2002). Several studies performed in various species have implicated Ebf genes in different steps and pathways of the nervous (Catela et al., 2019; Dubois and Vincent, 2001; Green and Vetter, 2011; Kratsios et al., 2012; Lin and Grosschedl, 1995) and the immune system development (Hagman et al., 1993), and their role as cancer treatment targets has recently been studied (Liao, 2009; Wang et al., 2021; Yao et al., 2024). Ebf2 is a member of this protein family and plays an essential role in nervous system development, especially during peripheral nerve myelination, neuron differentiation and migration, and cerebellar cortical topography determination (Badaloni et al., 2019; Chiara et al., 2012; Moruzzo et al., 2017; Wang et al., 2004). Various studies have also shown that Ebf2 is linked to peripheral neuropathy, including axonal damage and impaired motor nerve conduction (Giacomini et al., 2011), as well as the development of the neuroendocrine axis (Corradi et al., 2003). Outside the nervous system, Ebf2 is an essential transcriptional regulator for the determination of brown fat cell fate and bone metabolism (Kieslinger et al., 2005; Liao et al., 2024; Rajakumari et al., 2013; Wang et al., 2014) and is required for the proper stromal support of hematopoietic progenitor cells (Kieslinger et al., 2010).

Various studies conducted on Ebf2-null mutant mice have reported distinct phenotypic traits. These null-mutant animals show decreased numbers of orexin-producing cells in the lateral hypothalamus (LH), which are related to a narcolepsy-cataplexy phenotype (De La Herrán-Arita et al., 2011). On the other hand, it has been demonstrated that loss of Ebf2 caused a marked reduction in the number of dopaminergic (DA) neurons in the midbrain ventral periaqueductal gray matter (PAG) (Yang et al., 2015). These DA neurons play a crucial role in sleep–wake regulation, because they are active during wakefulness and suppressing their activity leads to an increase in daily sleep duration (Shaw et al., 2010). Moreover, Ebf2-null mice present several anatomical alterations, such as an overall reduction in brain size (Chiara et al., 2012), reduced bone mass (Kieslinger et al., 2005), and dwarfism. These mice also present defective migration of gonadotropin releasing hormone-synthesizing neurons, leading to secondary hypogonadism and infertility (Corradi et al., 2003), locomotor impairment, deficient motor coordination and motor learning, correlated to an ataxic gait phenotype (Badaloni et al., 2019; Hoxha et al., 2013).

While recent progress has illuminated the role of Ebf2 in the cerebellum and olfactory system, its early postnatal expression patterns and functional contributions to broader brain development remain poorly characterized. Our study addresses this gap by detailing the postnatal expression of Ebf2 across subcortical, midbrain, and hindbrain regions. Using the histological marker tau-GFP (TGFP) under the control of the Ebf2 promoter, we identified high expression levels within the motivation/reward and pain-modulating systems, as well as in areas associated with sensory and motor processing. Furthermore, a comparative analysis between Ebf2-null and hemizygous (Ebf2+/−) genotypes revealed significant expression differences in several of these regions. These findings offer new insights into the potential role of Ebf2 in regulating behavioral patterns, pain processing, and sensorimotor systems.

## 2 Materials and methods

### 2.1 Animals

We conducted all experiments in accordance with the Mexican Official Standard NOM-062-ZOO-1999 for the production, care, and use of laboratory animals. The Animal Care Committee of the University of Monterrey approved all procedures. We housed all mice in standard transparent polysulfone cages, in rooms equipped with ventilation, suction, and filtration systems, and automatic lighting. They were kept under standard conditions at 22–25 °C with a relative humidity of 40–60%, and a 12-h light/dark cycle with access to filtered water and food *ad libitum*.

We used the genetic histological marker green fluorescent protein (GFP) fused to the axonal tau protein (TGFP), expressed under the control of the Ebf2 gene promoter, in genetically modified mice produced as previously described (Wang et al., 2004). In brief, the first five exons of the coding region of the Ebf2 gene were replaced with the coding region for the fusion protein tau-green fluorescent protein (TGFP) using directed gene targeting techniques, resulting in the 129Sv*^Ebf2 − TGFP^* mouse strain. In hemizygous EBF2-TGFP (Ebf2+/−) mice, we use the GFP fluorescent signal to track Ebf2 expression, and the fusion with the axonal protein tau distributes the signal along the axons of Ebf2 cells. Homozygous Ebf2-TGFP (Ebf2−/−) animals lack the activity of Ebf2 and are thus null-mutant (KO) mice for this transcription factor. We observed no phenotypic differences between Ebf2+/− and Ebf2+/+ littermates, consistent with previous reports (Chiara et al., 2012). In this study, we used Ebf2+/− mice as controls and compared them to Ebf2−/− mice. We performed all experiments on 10-day-old (P10) male mice, using a total of six animals, three for each genotype. These were determined by means of PCR using DNA isolated from mouse tails, using the ADN Wizard Genomic kit (Madison, WI, USA) in accordance with the manufacturer’s instructions.

### 2.2 Brain slicing

Mice were deeply anesthetized using sodium pentobarbital administered intraperitoneally. Under deep anesthesia, the mice were perfused intracardially through the left ventricle with 30 mL phosphate-buffered saline (PBS, pH 7.4), followed by 4% paraformaldehyde (PFA) in PBS (50 mL, pH 7.4, Sigma-Aldrich) and 30 mL of 1X PBS. Following perfusion, the brains were removed from the skull and postfixed overnight in PFA at 4 °C. They were then cryoprotected in 20% sucrose in 1X PBS (pH 7.4) until sectioning. Finally, the brains were sectioned using a cryostat microtome (Leica CM1860 UV) at −25 °C into sagittal, horizontal, and coronal sections of 50 μm thickness. We trimmed the block until reaching the brain region from which we obtained the sections. Slices were mounted as consecutive series on APEX SAS® (Leica) slides and stored at −80 °C for further processing.

### 2.3 Immunohistochemistry

The brain slices were dried in an incubator at 37 °C for 30 min, then rehydrated and rinsed four times with a blocking solution containing 0.1 Tween-20, 0.03 M glycine in 1x PBS (PBSTG). Sections were then blocked for 1 h with a 5% normal donkey serum, 0.3% Triton-X in 1x PBSTG solution at room temperature. Next, sections were incubated at 4° C for 48 h in primary antibody solution (Chicken Anti-GFP, cat. 600-901-B12, Rockland Immunochemicals, PA, USA) at 1 μg/mL in PBSTG, covered with Parafilm®. Following this, the slices were rinsed three times in PBS-Tween. After rinsing, sections were incubated for 2 h with a secondary antibody solution (Alexa Fluor® 647 AffiniPure F(ab’)2 Fragment Donkey Anti-Chicken IgY, cat. 703–546-155, Jackson Immunoresearch, PA, USA) at 2 ng/mL in PBST at room temperature, in a humid chamber. Sections were rinsed 2x in PBST and 1x in PBS and then were mounted in Fluro-Gel II® with DAPI (Electron Microscopy Sciences, cat. 17,985-50, PA, USA) and left to dry for 1 h to allow the mounting solution to gel.

### 2.4 Image processing

Brain slice images were acquired using a Zeiss Axio Imager 2 fluorescent microscope and Zen PRO 3.1 software, at a 25 × oil immersion objective, automatically stitched with 10% overlap. Gamma correction and histogram modification were also applied to increase the contrast between the fluorescent signal and the image background. All images were saved at a depth of 8 bits, in PNG format, and down sampled to 1.82 μm/pixel resolution for faster workflow.

To achieve a 3D visualization of the Ebf2 expression pattern, the stack of image slices was first aligned using the *StackReg* ImageJ plug-in in rigid-body mode, restricting adjustments to rotations and translations. Following alignment, image registration was performed using the Allen Mouse Brain Atlas (Allen Institute for Brain Science, 2011). as a spatial reference. The images were then separated into discrete channels: blue for DAPI and green for the TGFP signal. To reduce noise, Median and Gaussian filters were applied to each channel.

**Figure 1:**
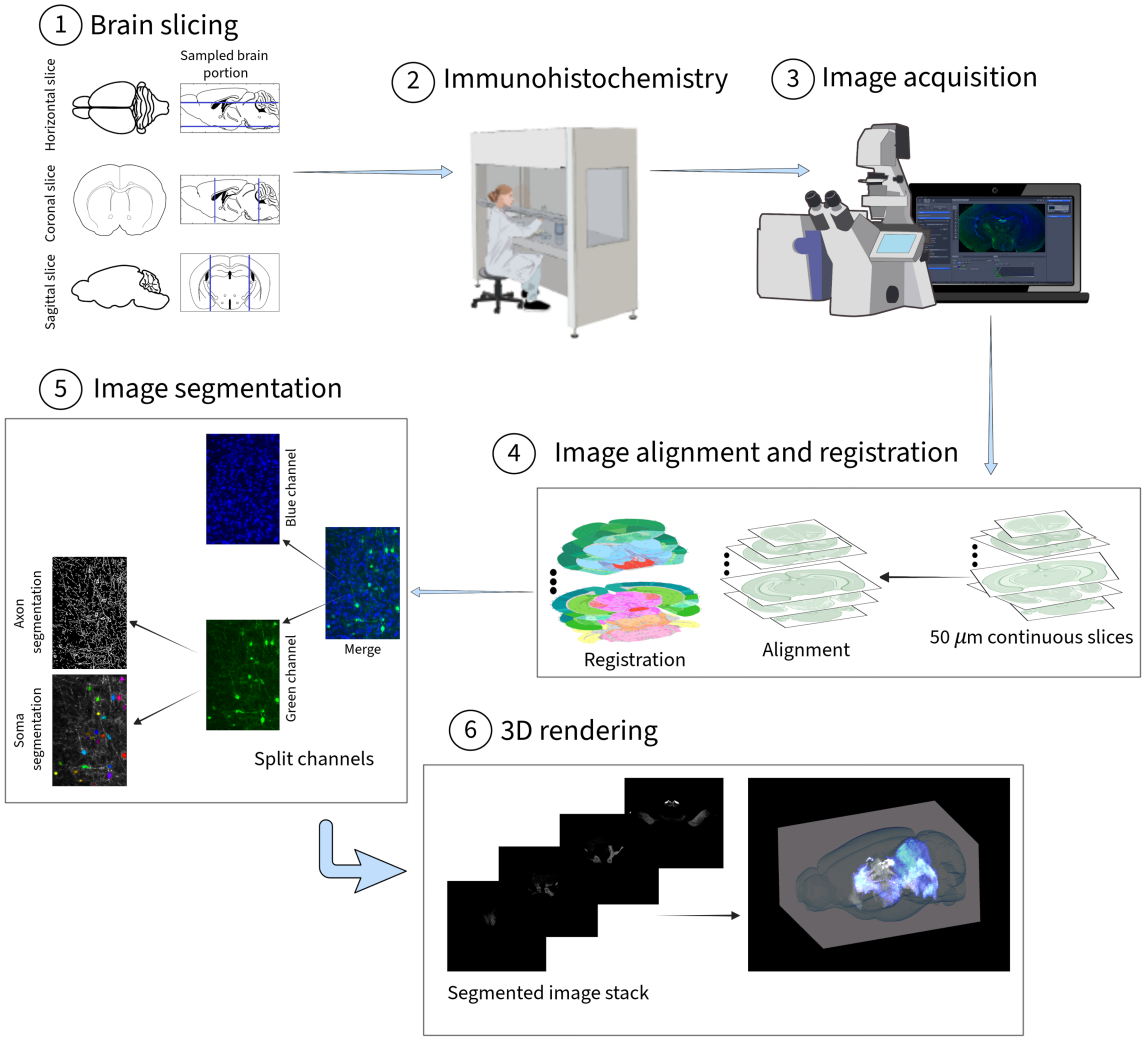
Overview of the methodology. (1) The brains were sectioned into sagittal, horizontal, and coronal sections of 50 μm thickness. (2) Secondary antigen immunostaining was applied to recover the GFP fluorescent signal. (3) Brain slice images were acquired using a fluorescent microscope. Illustration from NIAID NIH BioArt Source (bioart.niaid.nih.gov/bioart/, accessed in March 2026). All images were saved at a depth of 8 bits, in PNG format, and down sampled to 1.82 μm/pixel resolution. The green channel was assigned to capture the Tau-GFP signal, and the blue channel was used for capturing the DAPI signal. (4) Alignment and registration of images to the Allen Mouse CCFv3 reference atlas (atlas.brain-map.org, accessed in March 2025). (5) The images were separated by channels: green channel images were used for soma and axon segmentation, while blue channel images were used for nucleus segmentation and counting. (6) The stack of segmented images was rendered for 3D visualization using Napari.

Somata and axons were segmented using the green channel images. Soma segmentation was conducted via *Cellpose*, a deep learning U-Net algorithm (Stringer et al., 2021) that segments cells based on morphological features rather than intensity thresholds. The cyto3 model was used. The number of Ebf2-positive cells was subsequently quantified for each slice. For axon segmentation, an automated tool based on the eigenvalues of the Hessian matrix was developed to enhance and detect tubular structures based on local contrast gradients, and applied to the green channel grayscale images. The resulting segmented images were manually refined to remove false positives, such as background pixels incorrectly identified as somas or axons. Finally, 3D rendering was performed using *Napari*, a Python-based visualization tool (Chiu and Clack, 2022) (see Figure 1).

TGFP fluorescence intensity was quantified using Fiji/ImageJ. Background correction was performed using the Rolling Ball algorithm with a radius of 50 pixels. Regions of interest (ROI) were manually defined. Fluorescence intensity was measured as *Integrated Density*, defined as $\text{Integrated Density}=\mathrm{Are}a_{\mathrm{ROI}}\times \text{Mean Intensit}y_{\mathrm{ROI}}$ .

### 2.5 Statistical analysis

All statistical analyses were conducted in R (version 4.4.0; R Core Team, 2024) using the packages lme4, lmerTest and dplyr. Quantification of Ebf2-TGFP–positive cells was performed on multiple serial brain sections obtained from three animals per genotype (heterozygous and homozygous). Individual sections were therefore treated as repeated measurements rather than independent biological replicates. All inferential analyses used linear mixed-effects models. For each brain region, the number of Ebf2-TGFP–positive cells per section was modeled with genotype as a fixed effect and animal identity as a random intercept. This approach accounted for the nested structure of sections within animals and avoided pseudoreplication. To correct for multiple comparisons across brain regions, *p*-values were adjusted using both the Bonferroni and Benjamini–Hochberg false discovery rate (FDR) procedures. Statistical significance was set at *p* < 0.05. Differences in TGFP fluorescence intensity between genotypes were evaluated using a Mixed Linear Model in the same way. Statistical significance was set at *p* < 0.05.

## 3 Results

To ensure high confidence in the identification of Ebf2-expressing structures and to minimize false positives, an inclusion criterion was implemented: an anatomical region was designated as Ebf2-positive only if the TGFP signal was consistently observed in all three histological planes (coronal, sagittal, and horizontal). Although the Ebf2 signal was detected in caudal portions of the medulla oblongata, such as the spinal trigeminal nuclei, in sagittal sections; these regions were excluded from the final 3D reconstruction if cross-validation across all planes was not possible due to technical limitations in tissue processing at the extreme caudal-most levels. Therefore, the present dataset provides a high-certainty map of Ebf2 expression extending rostro-caudally from the septum up to the vestibular nuclei in the medulla oblongata.

### 3.1 Ebf2 is expressed in the motivation and reward system

**Figure 2:**
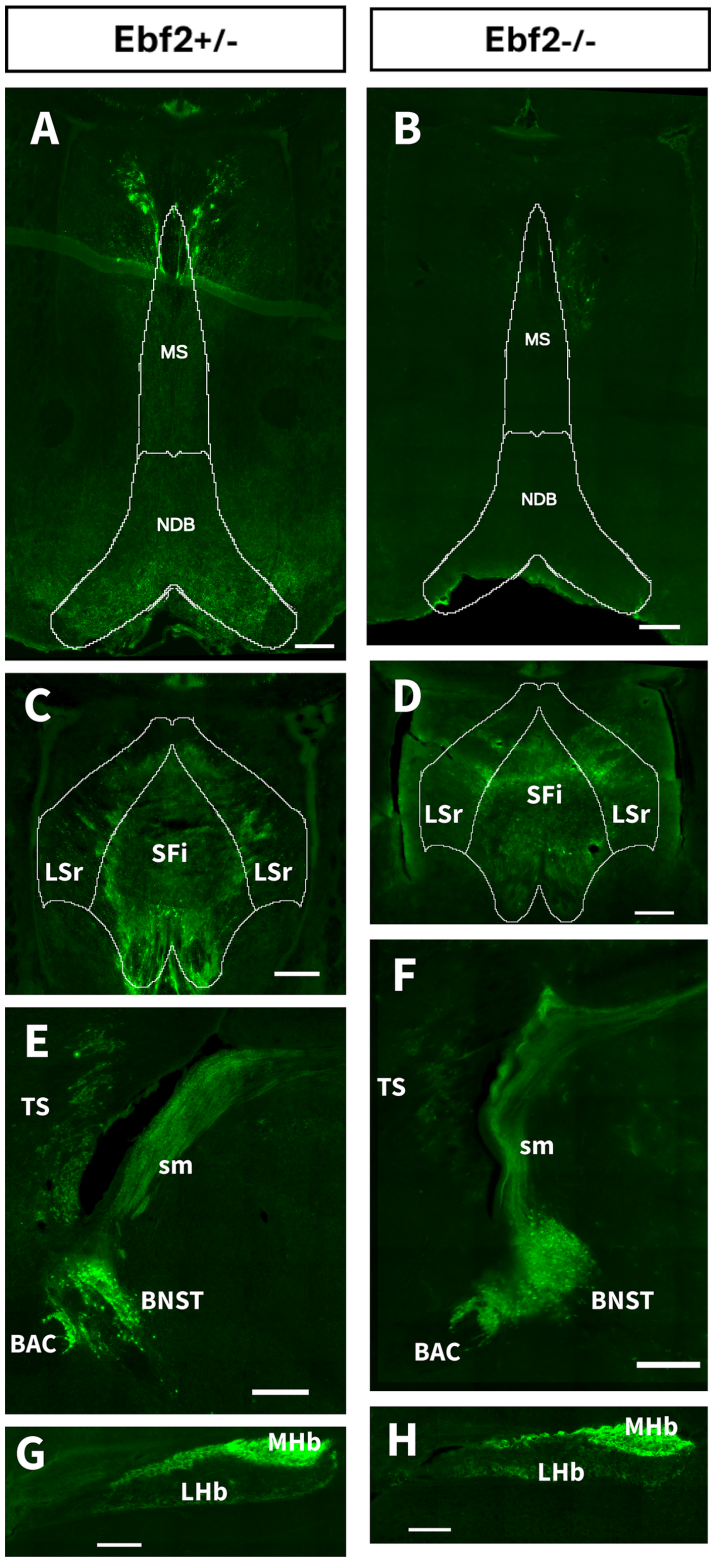
Representative images of Ebf2-TGFP expression in the septal and habenular region of hemizygous (Ebf2+/−) and null-mutant (Ebf2−/−) animals. **(A–D)** Coronal sections. **(E–H)** Sagittal sections. Scale bar: 200 μm. BAC, bed nuclei of the anterior commissure; BNST, bed nuclei of the stria terminalis; LHb, lateral habenula; LSr, lateral septal nucleus; MHb, medial habenula; MS, medial septal nucleus; NDB, nucleus of the diagonal band; SFi, septo fimbrial nucleus; sm, stria medullaris; TS, triangular septal nucleus. Scale bar: 200 μm.

**Figure 3:**
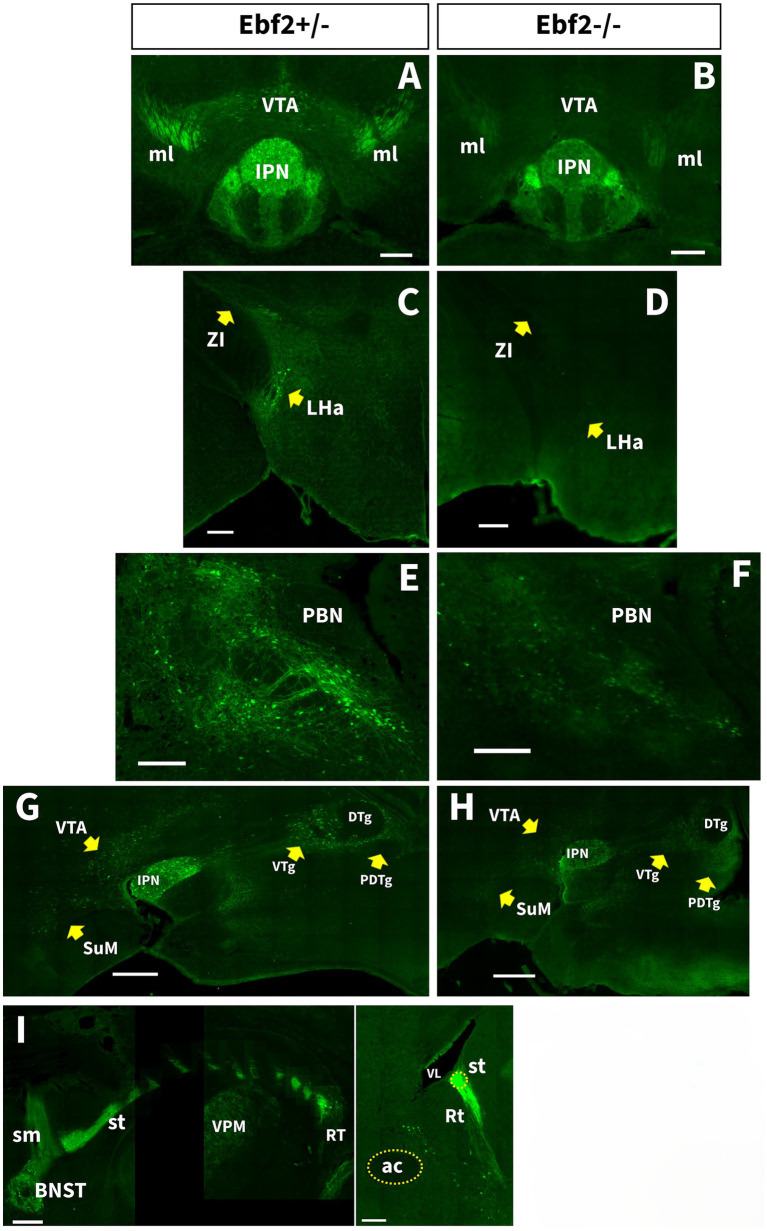
The number of TGFP cell populations is decreased in Ebf2−/− mice. **(A,B)** Coronal sections at the level of the IPN and VTA. Scale bar: 200 μm. **(C,D)** Coronal sections at the level of the LHA and ZI. Scale bar: 200 μm. **(E,F)** Coronal sections of the PBN. **(G,H)** Representative sagittal sections of the previous regions. Scale bar: 500 μm. **(I)** Left: consecutive coronal sections following the rostro caudal trajectory of TGFP axons arising from the BNST and projecting to the reticular thalamic nucleus (Rt) via the stria terminalis (st) in Ebf2+/− mice. Right: sagittal view of the st connecting with Rt. Scale bar: 200 μm. ac, anterior commissure; DTg, dorsal tegmental nucleus; IPN, interpeduncular nucleus; LHa, lateral hypothalamic area; ml, medial lemniscus; PBN, parabrachial nucleus; PDTg, posterodorsal tegmental nucleus; Rt, reticular thalamic nucleus; st, stria terminalis; SuM, supramammillary region of the hypothalamus; VL, lateral ventricle; VPM, ventral posteromedial nucleus of the thalamus; VTA, ventral tegmental area; VTg, ventral tegmental nucleus; ZI, zona incerta.

We found that Ebf2 is expressed in regions that comprise the dorsal diencephalic conduction system (DDC), as well as other areas involved in motivation and reward behavior. Both Ebf2+/− and Ebf2−/− mice showed TGFP signals in the septal nuclei (“septum”). This area is divided into four regions based on their anatomical location: the lateral, medial, posterior, and ventral groups (Risold, 2004). In Ebf2+/− mice, many axons were found in the medial septal complex [medial septal nucleus (MS) and nucleus of the diagonal band (NDB)] (Figure 2A), while in Ebf2−/−, the axon density appears to be lower (Figure 2B). The lateral septal nucleus (LSr) and the septofimbrial nucleus (SFi), which belong to the lateral group of the septum (LS), showed Ebf2-TGFP neurons, both in hemizygous and Ebf2-null mutant mice (Figures 2C,D). The posterior region [the triangular septal nucleus (TS) and the bed nuclei of the anterior commissure (BAC)] and the ventral septum [bed nuclei of the stria terminalis (BNST)] also exhibited the presence of Ebf2 cells in both genotypes (Figures 2E,F). The axons of the latter project to the medial habenula (MHb) and lateral habenula (LHb) regions via the stria medullaris (sm) (Figures 2G,H). Finally, Ebf2 expression was found along the stria terminalis (st), which connects the medial and central amygdala with the BNST and other hypothalamic regions (Figure 3I).

Other important regions that process information about motivation and reward behavior, where TGFP cell bodies and axonal fibers were found in Ebf2+/− mice, are the lateral hypothalamic area (LHa), the zona incerta (ZI, Figures 3C,D), the ventral tegmental area (VTA) and the interpeduncular nucleus (IPN, Figures 3A,B,G,H). Remarkably, the Ebf2-TGFP axons that innervate the IPN do not follow the prototypical habenula-fasciculus retroflexus pathway, which is part of the DDC circuit. Instead, these axons originate from Ebf2-TGFP cells located in the pontine ventral tegmental (VTg) and central gray (PCG) areas that surround the dorsal (DTg) and posterodorsal tegmental nucleus (PDTg) (Figures 3G,H). In addition, we observed Ebf2-TGFP cells in regions that act as descending targets or ascending modulators of these motivation and reward systems, such as the parabrachial nucleus (PBN, Figures 3E,F) and the supramammillary region of the hypothalamus (SuM, Figures 3E,F).

**Figure 4:**
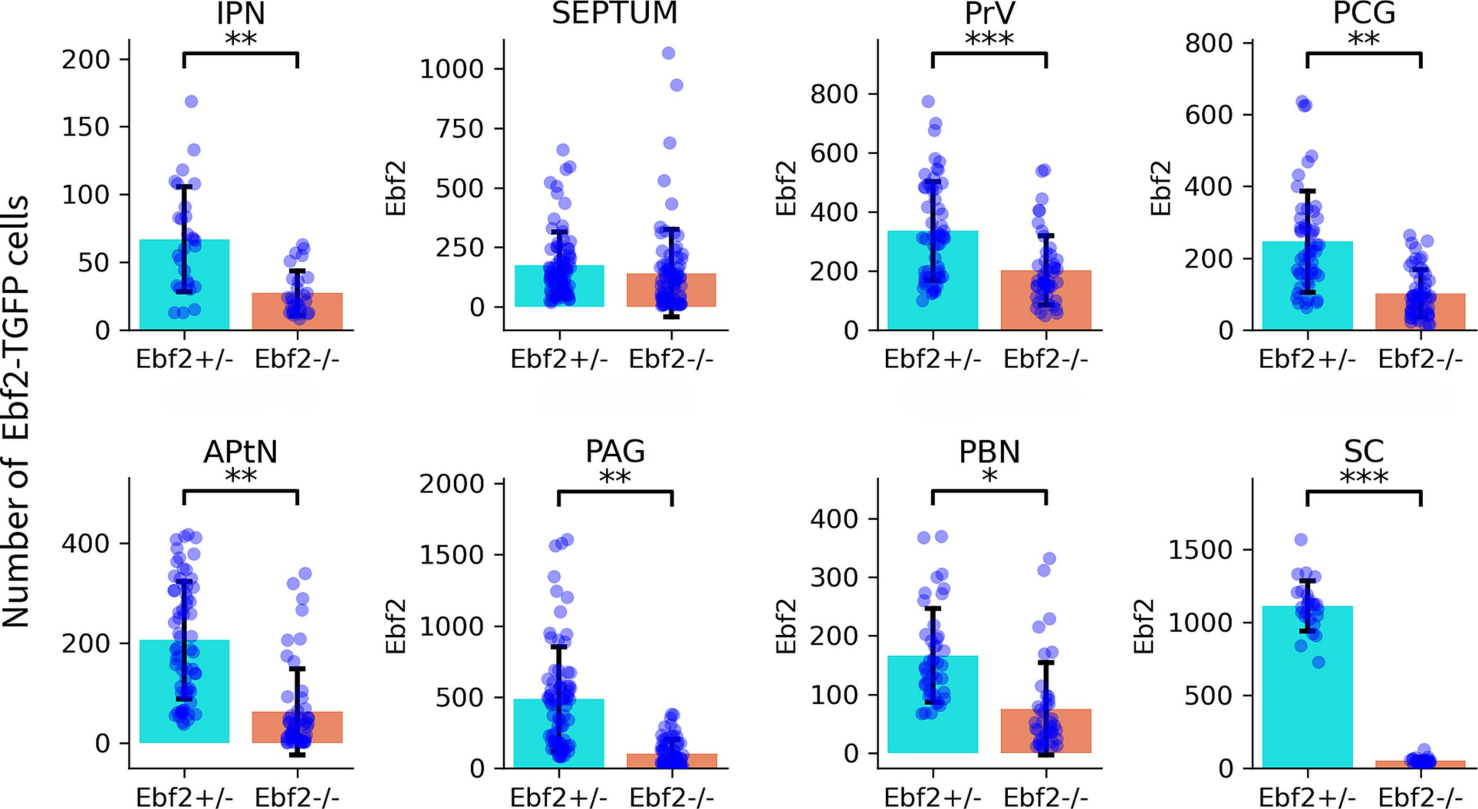
Ebf2-TGFP positive cells were quantified across multiple brain regions in Ebf2+/− and Ebf2−/− mice. Each dot represents the number of Ebf2-TGFP–positive cells quantified in a single brain section. Three animals per genotype were analyzed (*n* = 3), with multiple serial sections collected from each mice. Bars indicate the mean ± standard deviation. Statistical comparisons between genotypes were conducted using linear mixed-effects models, with genotype as a fixed effect and animal identity as a random effect. *p*-values were adjusted for multiple comparisons across regions using Benjamini–Hochberg FDR method. Significance levels: *p < 0.05, **p < 0.01, ***p < 0.001.

Quantification of Ebf2-TGFP somata was conducted in the components of the motivation/reward circuits that possess relatively well-defined anatomical boundaries. Statistical analysis demonstrated significant decreases in Ebf2-TGFP cell number counts between hemizygous and null-mutant genotypes across all examined brain regions, except for the septal area regions (Figure 4).

### 3.2 Ebf2 is expressed in circuits associated with nociception

**Figure 5:**
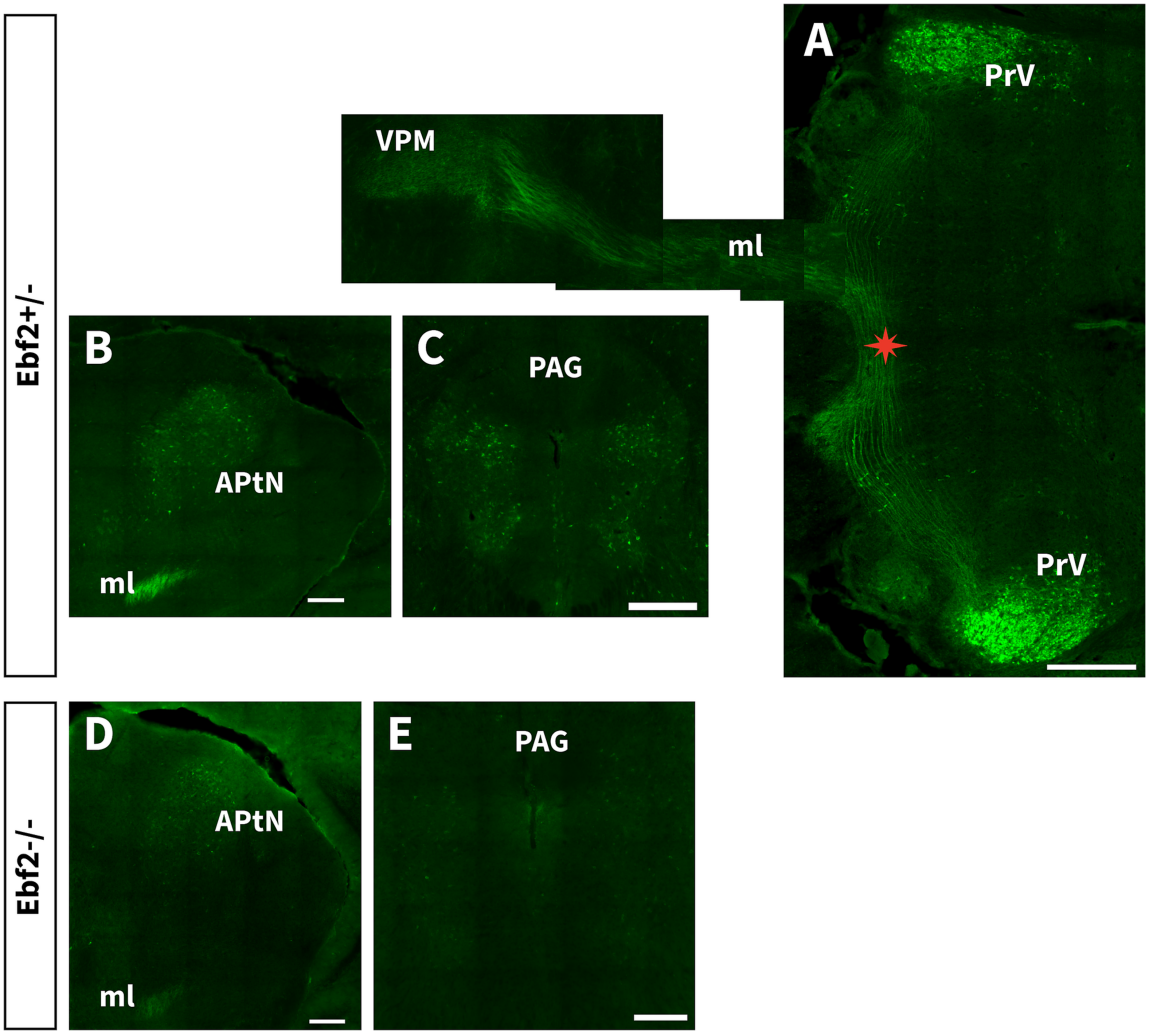
Ebf2 is expressed in the trigeminal lemniscus pathway and central nodes in the descending pain control system. **(A)** Consecutive oblique horizontal sections show Ebf2-TGFP axons arising from the PrV innervate and distribute diffusely across the VPM nucleus. Asterisk indicates the midline crossing (trigeminal lemniscus) of the PrV axons. Scale bar: 500 μm. **(B–E)** Coronal sections of the APtN and PAG regions from hemizygous and null-mutant mice. Scale bar: 200 μm. APtN, anterior pretectal nucleus; ml, medial lemniscus; PAG, periaqueductal gray; PrV, principal sensory nucleus of the trigeminal; VMP, ventral posteromedial nucleus of the thalamus.

**Figure 6:**
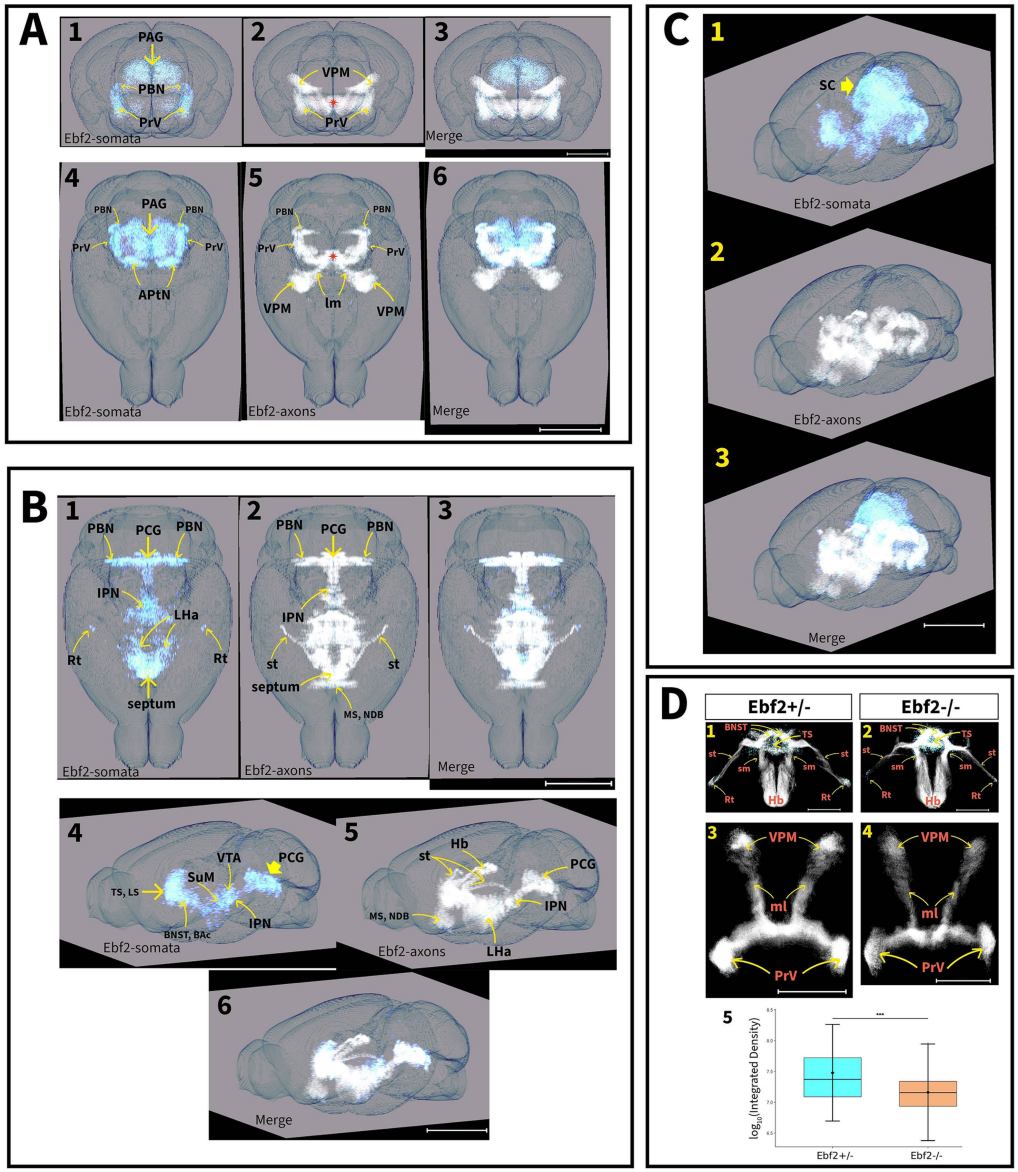
Tridimensional rendering of the composite expression pattern of Ebf2-TGFP signals. The cell bodies (somata, light blue) and axons (white) are shown separately. **(A)** Rendering of the Ebf2-TGFP signals in the nociceptive system of hemizygous mouse brains. A1-3: Coronal projections of the 3D model (scale bar: 1.5 mm). A4-6: Horizontal projections of the 3D model (scale bar: 2.5 mm). Asterisk indicates the location of the interpeduncular nucleus. **(B)** Rendering of the Ebf2-TGFP signals in the motivation/reward systems of hemizygous mouse brains. B1-3: Horizontal projection of the 3D model (scale bar: 2.7 mm). B4-6: Side view projection of the 3D model after a 30° rotation from the sagittal plane (scale bar: 3 mm). **(C)** Composite rendering of the full Ebf2-TGFP 3D-pattern of expression in the brain of hemizygous mice (scale bar: 3.2 mm). **(D)** Comparison of the composite 3D-models of the septal areas and PrV-VPM pathways from hemizygous and Ebf2-null mutant mice. D1-2: Horizontal projection of the septal and habenular areas (scale bar in D1: 1 mm; scale bar in D2: 900 μm). D3-4: Horizontal projection of the PrV-VPM pathways from hemizygous and Ebf2-null mutant mice (scale bar in D3: 1.8 mm; scale bar in D4: 1.3 mm). D5: Comparison of GFP fluorescence intensity of the PrV-VPM circuit between genotypes (***p < 0.001). APtN, anterior pretectal nucleus; BAC, bed nuclei of the anterior commissure; BNST, bed nuclei of the stria terminalis; Hb, habenula; IPN, interpeduncular nucleus; LHa, lateral hypothalamic area; MS, medial septal nucleus; ml, medial lemniscus; NDB, nucleus of the diagonal band; PAG, periaqueductal gray; PBN, parabrachial nucleus; PCG, pontine central gray; PrV, principal sensory nucleus of the trigeminal; Rt, reticular thalamic nucleus; SCs, superior colliculus; sm, stria medullaris; st, stria terminalis; SuM, supramammillary region of the hypothalamus; sm, stria medullaris; VMP, ventral posteromedial nucleus of the thalamus; VTA, ventral tegmental area; VTg, ventral tegmental nucleus; ZI, zona incerta.

We found Ebf2 expression in areas that integrate orofacial somatosensory information. Ebf2-TGFP is abundantly expressed in the principal sensory nucleus of the trigeminal nerve (PrV). A large bundle of axons crosses the midline from the PrV in parallel arrays. It makes a sharp rostral turn immediately after crossing the midline (Figure 5A). Then it ascends with the medial lemniscus (ml), ultimately reaching the ventral posteromedial nucleus of the thalamus (VPM). No differences in connectivity were observed between genotypes along this pathway, indicating that its fundamental organization is preserved even in the absence of Ebf2. However, Ebf2−/− mice showed a significant reduction in Ebf2-TGFP cells (Figure 4), as well as lower axonal density (Figures 6D2,D4). TGFP fluorescence intensity was compared in the PrV-VPM circuit. Mixed linear model analysis demonstrated a significant genotype effect on TGFP fluorescence intensity ( $p=1.1\times 10^{-9}$ ). Ebf2−/− mice exhibited an approximately 50% reduction in TGFP signal relative to Ebf2+/− mice, after accounting for variability due to brain slice type (Figure 6D5).

Another key region of the nociceptive system where Ebf2-TGFP neurons can be detected is the parabrachial nucleus complex (PBN, Figures 3E,F). Moreover, in Ebf2+/− mice, a large number of Ebf2 somata were observed within two regions that play an important role in the processing of nociceptive signals: the periaqueductal gray (PAG) and the anterior pretectal nucleus (APtN, Figures 5B–E). Statistical analysis revealed a significant decrease in the number of TGFP cells in Ebf2-null mutant animals in these brain regions (Figure 4).

### 3.3 Ebf2 is expressed in the superior colliculus and several thalamic nuclei

**Figure 7:**
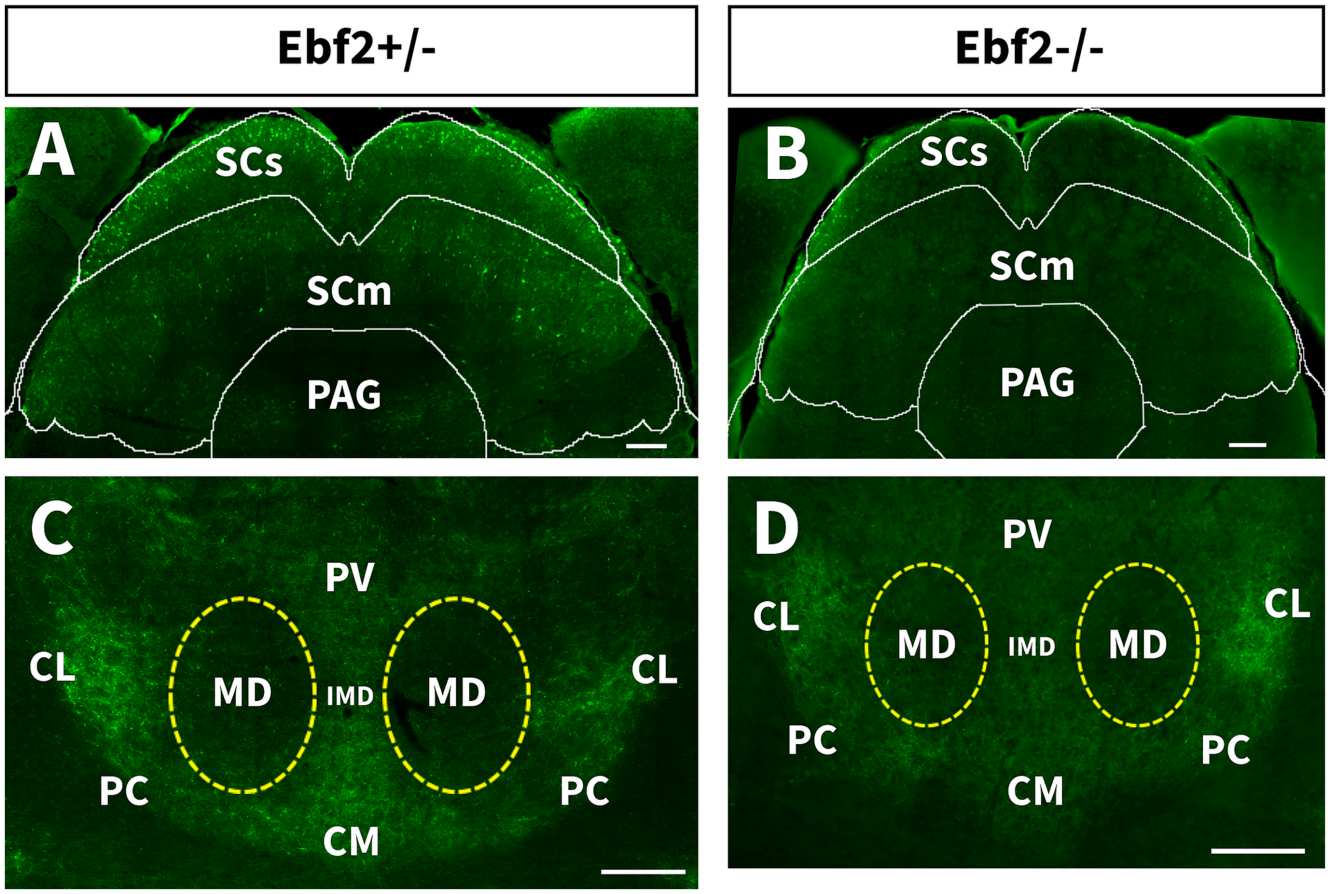
Localization of Ebf2-TGFP signals in the superior colliculus and thalamic nuclei. **(A,B)** Coronal sections of superior colliculus (SC). Scale bar: 200 μm. **(C,D)** Coronal sections of intralaminar nuclei of the thalamus (ILt). Scale bar: 200 μm. CM, central medial nuclei; CL, central lateral nuclei; IMD, intermediodorsal nucleus of thalamus; MD, mediodorsal nucleus of thalamus; PAG, periaqueductal gray; PC, paracentral nuclei; PV, paraventricular nuclei of the thalamus; SCs, sensory area of the superior colliculus; SCm, motor area of the superior colliculus.

In Ebf2+/− mice, a large number of TGFP cell bodies is found in the superior colliculus (SC), both in sensory (SCs) and motor (SCm) areas. Corresponding to our previous findings, in Ebf2-null mice, the number of TGFP-positive cells is drastically reduced in this region (Figures 4, 7A,B).

Furthermore, we found Ebf2 axons in the intralaminar nuclei of the thalamus (ILt), which appear as Y-shaped in coronal views and are divided into rostral and caudal divisions. We found TGFP signals in the rostral group consisting of the central medial (CM), central lateral (CL), and paracentral (PC) nuclei, in addition to the interomediodorsal (IMD) and paraventricular (PV) nuclei of the thalamus (Figures 7C,D). These signals are decreased in Ebf2-null mutant mice.

Finally, some axons originating from Ebf2 neurons in the BNST travel along the stria terminalis (st) and end up projecting to the reticular (Rt) nucleus of the thalamus, in a region located more dorsal and more lateral to the VPM nucleus (Figure 3I). Some Ebf2-TFP cell bodies can also be found in the Rt nucleus. This BNST-Rt circuit appears to be similar in both hemizygous and Ebf2-null mice.

Figure 6 shows the three-dimensional composite average reconstruction of the Ebf2-TGFP expression pattern, including both somata and axons, in the brains of P10 mice. This reconstruction is based on three coronal section stacks and has been registered to the Allen Institute’s brain atlas, which is widely regarded as the gold standard for three-dimensional mouse brain reconstruction. The nociception system (Figure 6A), the motivation and reward system (Figure 6B), and the complete model (Figure 6C) are displayed separately. Given the established role of Ebf2 in neuronal development, we expected to observe drastic changes in connectivity between hemizygous and Ebf2-null mutant mice. However, both the overall expression pattern and the organization of connections are preserved between the two genotypes, although the null mutants show decreased numbers of TGFP somata across multiple regions of the brain.

## 4 Discussion

Current research reveals an important role for the transcription factor Ebf2 in the integrity of multiple brain circuits in mice, with implications for motivation and reward systems, as well as nociception and various sensory processing circuits.

### 4.1 Motivation and reward system

Our results show that Ebf2 is expressed in multiple nodes of the motivation and reward circuits of the brain, including septal, hypothalamic, tegmental, and pontine structures. This widespread distribution suggests that Ebf2 not only participates in neuronal differentiation, as previously described, but could also be decisive in establishing functional networks that link motivational states with goal-directed behavior.

We found an abundant expression of Ebf2 in the septal-habenular-tegmental circuit, a fundamental axis in the encoding of aversive and reward signals (Li et al., 2021; Zhang et al., 2018). The septal axons project to MHb and LHb via the sm, and these habenular structures in turn modulate dopaminergic activity in the VTA (Ji and Shepard, 2007; Matsumoto and Hikosaka, 2007; Wallace et al., 2020). Usually, the MHb neurons send most of their efferent projections to the IPN through the fasciculus retroflexus (fr), which, in turn, sends efferent and afferent projections to a wide variety of structures implicated in motivation and reward circuitry, which include DTg, PDTg, VTA, septum and LHb (Roman et al., 2020; McLaughlin et al., 2017).

Ebf2-TGP axons originating in the septal nuclei follow the sm into the habenular areas but no Ebf2-TGP somata can be detected in either the MHb or the LHb. This finding correlates with the absence of TGFP axons within the fr of hemizygous or Ebf2-null mutant animals. However, in animals belonging to both genotypes, a dense innervation of the IPN can be observed, from axons originating from neurons located in the pontine VTg nucleus and central gray areas (PCG). These circuits integrate the valence of sensory information required to modulate the motivation/reward output and are involved in regulation of arousal state and addictive behaviors (Xiao et al., 2023).

In these septal-habenular and pontine-IPN circuits, no substantial decreases in TGFP density is detected in Ebf2-null mutant animals (Figures 2E–H, see also 6D1 and 6D2). As Ebf2 has early developmental roles in the septum (Chiara et al., 2012), residual expression or compensation by other Ebf isoforms (e.g., Ebf3) may prevent significant loss at the analyzed age. However, we observed a significant decrease in TGFP cell numbers in the LHA, ZI, and VTA of Ebf2-null mutant mice. These regions are recognized as centers that generate motivation and reward signals that regulate behavior, as they integrate homeostatic information with mesolimbic dopaminergic activation (Ye et al., 2023; Yousefvand and Hamidi, 2022). The presence of Ebf2 in these regions in hemizygous animals and its reduction in Ebf2-null mice leads to the hypothesis that this transcription factor ensures connectivity between homeostatic systems and dopaminergic circuits, allowing for the adequate translation of physiological states into arousal state and/or motivated behaviors.

Correlating with these latter findings, it has been shown that Ebf2-null mice have a significant decrease in orexinergic neurons in the LHa (De La Herrán-Arita et al., 2011), affecting the regulation of wakefulness. In this context, also there is a functional relationship between the LHa and the Rt nucleus of the thalamus, which is principally inhibitory (Guillery and Harting, 2003), and recent evidence has identified orexinergic neurons within the Rt which participate in the regulation of the sleep–wake cycle in rats (Morina and Romanova, 2022). On the other hand, recent studies have shown that stimulation of PBN induces states of wakefulness, mediated by glutamatergic neurons (Qiu et al., 2016; Xu et al., 2021), but it is also associated with modulation of feeding behavior through reward pathways, including orexigenic regulation of feeding (Zhao et al., 2023).

Furthermore, in recent years, it has been shown that SuM glutamatergic neurons project to MS glutamatergic neurons and are responsible for modulating both the motivation for environmental interaction and wakefulness (Kesner et al., 2021; Liang et al., 2023). Finally, it has been demonstrated that the loss of Ebf2 results in a significant decrease in dopaminergic (DA) neurons in the PAG (Yang et al., 2015). Although this region is mainly associated with pain modulation, DA neurons play an important role in sleep–wake regulation (Zhang et al., 2024). Thus, the decreased number of TGFP cells in these structures observed in Ebf2-null mutant animals leads to the idea that Ebf2 may be necessary for the maintenance of functional dopaminergic, orexinergic, and glutamatergic circuits, jointly regulating wakefulness, arousal, motivation, and reward-seeking behavior.

Taking together, our results suggest that Ebf2 plays distinct roles across different brain regions: it is important for the organization and connectivity of midbrain and hypothalamic nuclei that are part of the motivational/reward systems but appears to be dispensable for maintaining cell density in the septum. It remains to be studied if the TGFP septal neurons present in Ebf2-null mutant mice preserve their differentiation identity.

### 4.2 Pain perception and modulation

Previous studies have documented the expression of Ebf2 in the principal sensory nucleus of the trigeminal nerve (PrV) during embryonic stages (Ding et al., 2003). Our current results reveal that Ebf2 continues to be expressed during postnatal stages. The main finding is that the overall pathway of neural connectivity between the PrV and the VPM nucleus of the thalamus is unchanged in Ebf2-null mutant mice compared with hemizygous animals. However, there seems to be a lower axonal density in the PrV-VPM circuit in Ebf2-null mutant mice (Figures 6D3,D4), possibly due to the decrease in the number of Ebf2-TGFP cells in the PrV of null-mutant animals or a possible reduction in axonal caliber, as observed in peripheral nerves in Ebf2−/− mice (Giacomini et al., 2011). This proper neural connectivity observed in Ebf2-null mutant animals indicates that Ebf2 is not essential for the establishment of tactile discriminative connections. However, the observed reduction in Ebf2-TGFP signal intensity in the null-mutant mice suggests a functional decrease in the involvement of Ebf2 neurons in somatosensitive processing.

The almost complete loss of Ebf2-TGFP neurons in the PAG and APtN in Ebf2-null mutant mice is particularly relevant. The PAG is a central node in the descending pain control system, modulating nociceptive transmission at the spinal level through projections to the rostroventromedial medulla (Hemington and Coulombe, 2015; Keay and Bandler, 2015; Zhang et al., 2024), and it has recently been revealed that the circuit of orexin neurons from LHa to PAG regions serves in the contrasting modulation of itch and pain processing (Kaneko et al., 2024). This fact is interesting because the loss of Ebf2 causes a decrease in the number of orexinergic neurons in the LHa, as described above. For its part, the APtN has been implicated in analgesia mechanisms and in the inhibition of nociceptive reflexes (Genaro and Prado, 2021). The fact that the loss of Ebf2 has such a significant impact on neurons in the LHa, PAG, and APtN suggests that it could act as a key transcriptional regulator for the assembly of modulatory sensory LHa–PAG-AtPN circuit, which integrate internal signals (such as wakefulness or metabolism, modulated by orexin) with peripheral sensory perception (pain, itching), predisposing to a phenotype of hyperalgesia in the Ebf2-null mutant mice.

### 4.3 Ebf2 expression in the superior colliculus and innervation of several thalamic nuclei

We observed Ebf2-TGFP innervation of several thalamic structures, including the intralaminar (CM, CL, PC), interomediodorsal (IMD), and paraventricular (PV) nuclei, which is preserved Ebf2-null mutant mice when compared to hemizygous animals, although with reduced axonal density in the former group. The intralaminar nuclei of the thalamus are involved in modulation of arousal, cognition, pain processing and proprioception, affective behaviors, motivation, and reward-related processes (Kato et al., 2018; Kumar et al., 2023; Vertes et al., 2022). The weak innervating TGFP signal present in Ebf2-null mutant mice suggests that Ebf2 may not be essential for the initial specification of the connectivity towards these thalamic nuclei, but it could participate in their functional maturation or synaptic refinement. Alternatively, other members of the Ebf family may be assuming functions in a redundant manner, as has been described in the development of the cerebellum and spinal cord (Badaloni et al., 2019; Croci et al., 2006; Garel et al., 1997).

In contrast, the superior colliculus (SC) shows a marked sensitivity to Ebf2 loss. In hemizygous mice, numerous positive cell bodies were detected in the sensory (SCs) and motor (SCm) regions of the colliculus, whereas in Ebf2-null mutant animals these populations are drastically reduced. The SCs layer is specialized for processing visual information, detecting salient stimuli, and integrating multisensory inputs (visual, auditory, somatosensory) to guide attention and perception (Benarroch, 2023; Benavidez et al., 2021; Liu et al., 2022; Rusciano and Bagnoli, 2025). The SCm layer receives input from SCs and multiple cortical and subcortical sources. These layers are responsible for transforming sensory signals into motor commands, initiating orienting movements (such as saccades, head, and body turns), and coordinating complex behaviors like escape or approach (Gandhi and Katnani, 2011; Liu et al., 2022; Rusciano and Bagnoli, 2025). The role that Ebf2 plays for the function of the SC circuits has not yet been systematically studied. However, evidence suggests that the OX_2_ receptor is predominantly expressed and functional in the SC, indicating that the orexinergic pathway in the SC is arousal-dependent (Chrobok et al., 2021). Given the available evidence on the influence of Ebf2 loss on the reduction of orexin-expressing cells, coupled with the fact that Ebf2-null mutant mice show a significant decrease in TGFP neurons in the SC, we can hypothesize that Ebf2 could be indispensable for the development or survival of specific subpopulations (possibly glutamatergic or GABAergic) that mediate the orexinergic response.

### 4.4 Cellular identity and developmental implications of Ebf2 expression

The cellular identity of the Ebf2-expressing population is characterized by an exclusively neuronal phenotype. Although Ebf2 is expressed in non-neuronal cells within the peripheral nervous system, transcriptomic studies of the developing mouse brain have demonstrated that Ebf2, together with other Ebf family members, specifically marks neuronal lineages progressing through a neuroblast state and is not expressed in glial lineages (La Manno et al., 2021).

On the other hand, Ebf2 is a well-established pioneer transcription factor essential for the development of various neuronal populations. For example, previous studies have established that the absence of Ebf2 leads to reduced expression of other markers such as Dbx1, which delineate the progenitor domains of the superior colliculus, and Reelin, signaling directs subsequent neuronal migration (Bielle et al., 2005; Chiara et al., 2012; Tran et al., 2024). Given that Ebf2 interacts with these migratory pathways in the cortex and cerebellum, the marked reduction of Ebf2-TGFP cells observed in the periaqueductal gray (PAG) and superior colliculus (SC) of Ebf2-null mice raises the question of whether this loss results from postnatal cell death or an earlier failure in neuronal differentiation and migration. Since the analysis was conducted at postnatal day 10 (P10), it is plausible that the reduction observed in the PAG and SC reflects a developmental defect established during embryogenesis. While the current data reliably map the outcome of this deficit, future lineage-tracing studies during embryonic stages are necessary to definitively distinguish between apoptosis and phenotype conversion in these specific nociceptive and reward circuits.

To elucidate the functional implications of the findings at P10, the known developmental roles of Ebf2 were systematically compared with its adult expression profile (Table 1). As indicated in Table 1, Ebf2 expression is not solely a developmental remnant; it persists into adulthood in specific regions, including the cerebellum, olfactory bulb, and, as demonstrated in this study, in the early postnatal motivation/reward, nociceptive, and somatosensory circuits. This sustained expression indicates a potential functional shift. While early expression is associated with morphogenesis and identity specification, postnatal and adult expression is likely necessary for circuit maintenance, plasticity, or the regulation of neurotransmitter phenotypes, such as dopaminergic identity in the PAG and orexinergic identity in the LHA, SC, and PBN. Our current P10 map, therefore, captures a transitional period in which developmental expression diminishes in certain areas, such as the cortex, while functional expression becomes established in others, such as the motivation/reward circuits.

**Table 1:** Expression pattern and role of Ebf2 in the mouse brain.

Brain structure*	Development stage	Developmental role	Effect of deletion/mutation	References
Forebrain (septum, cortical hem and the pallial–subpallial boundary)	E10.5-P0	Regulate early tangential migration from signaling centers (hem, septum) to the cortical surface.	Transient decrease in Cajal–Retzius (CR) cells on the cortical surface and delayed migration; partial compensation by Ebf3.	Chiara et al. (2012) and Chuang et al. (2012)
Cerebellum (cerebellar cortex and nuclei)	E10.5-E18.5; P20, P60.	Regulates the migration of Purkinje cell (PC) precursors from the ventricular zone (VZ) to the subependymal zone and of *Atoh1 +* precursors from the superior rhombic lip.	Reduction of number of PC and granular cells, and trans-differentiation from Zebrina II-negative to positive phenotype. Small cerebellum	Badaloni et al. (2019), Chung et al., 2008, and Croci et al. (2006)
Olfactory Bulb	E14, P56	Regulates neurogenesis and differentiation of sensory neurons from the olfactory epithelium.	Persists in the glomerular layer; involved in continuous neurogenesis and odorant receptor maintenance.	Wang et al. (2004)
APtN, PBN, ml PrV, VPM.	E12.5-E16.5; P10.	Participates in the differentiation of PrV neurons downstream of *Drg11*. Likely involved in the specification of nociceptive modulation.	Lower axonal density in the PrV-VPM circuit; potentially modulates pain thresholds.	Ding et al. (2003); Present study
Forebrain: Amygdala, BAC, BNST, Hb, LHa, Rt, sm, SuM, septum, VTA, ZI.	E10.5-P0; P10.	Likely necessary for the maintenance of functional dopaminergic, orexinergic, and glutamatergic circuits, jointly regulating wakefulness, arousal, motivation, and reward-seeking behavior.	Reduced expression of other markers, such as Reelin, Dbx1, and Ebf3. Reduced expression of orexin.	Chiara et al. (2012) and De La Herrán-Arita et al. (2011); Present study.
PAG, PCG, IPN, VTg.	P10	Likely marks a conserved circuits dedicated to coordinate survival responses to aversive stimuli	Significant reduction in Ebf2-TGFP cells.	Present study.
SCs, thalamic nuclei.	*P10*	Likely indispensable for the development or survival of specific neuron subpopulations.	Significant reduction in Ebf2-TGFP cells.	Present study.

*APtN, anterior pretectal nucleus; BAC, bed nuclei of the anterior commissure; BNST, bed nuclei of the stria terminalis; Hb, habenula; IPN, interpeduncular nucleus; LHa, lateral hypothalamic area; MS, medial septal nucleus; ml, medial lemniscus; NDB, nucleus of the diagonal band; PAG, periaqueductal gray; PBN, parabrachial nucleus; PCG, pontine central gray; PrV, principal sensory nucleus of the trigeminal; Rt, reticular thalamic nucleus; SCs, superior colliculus; st, stria terminalis; SuM, supramammillary region of the hypothalamus; sm, stria medullaris; VMP, ventral posteromedial nucleus of the thalamus; VTA, ventral tegmental area; VTg, ventral tegmental nucleus; ZI, zona incerta.

### 4.5 Limitations and future studies

One limitation of the present study is the absence of immunohistochemical co-labelling with neurotransmitter or functional markers, which precludes precise identification of the phenotype for all Ebf2-TGFP populations. Nevertheless, the global anatomical map established here offers a foundational framework for future targeted investigations. Further experiments during the embryonic development of Ebf2-null mutant mice are required to determine whether the observed reduction in cell number results from cell death or fate conversion. In addition, future work should determine if Ebf2 loss leads to measurable functional changes using behavioral assays for nociception, motivation, reward sensitivity, and arousal state. In addition, examining Ebf2 coexpression with specific neurochemical markers is necessary to clarify the molecular identity and functional diversity of Ebf2-expressing neurons within the motivation, reward, and nociceptive systems. Identifying whether Ebf2 is enriched in glutamatergic, GABAergic, or peptidergic neurons will provide critical insight into how this transcription factor influences the excitatory–inhibitory balance and neuropeptidergic modulation across circuits such as the parabrachial nucleus (PBN), lateral hypothalamic area (LHa), ventral tegmental area (VTA), and interpeduncular nucleus (IPN).

## 5 Conclusion

This study presents a detailed mapping of Early B-cell factor 2 (Ebf2) expression in the midbrain and hindbrain regions involved in motivation, reward, and pain modulation. Our data indicates that Ebf2, in addition to its established function as a developmental transcription factor, remains active during postnatal stages, particularly within neural circuits responsible for integrating sensory, motivational, and arousal signals. Observed reductions or absence of Ebf2-TGFP cells in specific nuclei of Ebf2-null mutant mice underscore its potential role in preserving the structural and functional integrity of these neural systems. Collectively, these results identify Ebf2 as a molecular regulator that coordinates dopaminergic, orexinergic, and glutamatergic pathways to modulate motivational states, pain processing, and wakefulness.

## Data Availability

The raw data supporting the conclusions of this article will be made available by the authors, without undue reservation.
